# Single-injection nerve blocks for total knee arthroplasty: femoral nerve block versus femoral triangle block versus adductor canal block—a randomized controlled double-blinded trial

**DOI:** 10.1007/s00402-023-04960-5

**Published:** 2023-06-30

**Authors:** Carlos I. Salvadores de Arzuaga, Marcos Miguel, Alfons Biarnés, Marcelo García, José Naya, Andrea Khoudeir, Joan Minguell, Oriol Pujol

**Affiliations:** 1 Orthopaedic Surgery Department, Vall d’Hebron University Hospital, Universitat Autònoma de Barcelona, Barcelona, Spain; 2 Anesthesiology and Reanimation Department, Vall d’Hebron University Hospital, Universitat Autònoma de Barcelona, Barcelona, Spain; 3 Physical Medicine and Rehabilitation Department, Vall d’Hebron University Hospital, Universitat Autònoma de Barcelona, Barcelona, Spain

**Keywords:** Randomized controlled trial, Total knee arthroplasty, Postoperative analgesia, Femoral nerve block, Femoral triangle block, Adductor canal block

## Abstract

**Introduction:**

Femoral nerve block (FNB) is a well-established analgesic technique for TKA. However, it associates quadriceps weakness. Therefore, femoral triangle block (FTB) and adductor canal block (ACB) were proposed as effective alternative motor-spearing techniques. The primary objective was to compare quadriceps muscle strength preservation between FNB, FTB and ACB in TKA. The secondary objective was to analyze pain control and functional outcomes.

**Methods:**

This is a prospective, double-blinded RCT. From April 2018 to April 2019, patients who undergo a primary TKA were randomized into three experimental groups: FNB-G1/FTB-G2/ACB-G3. Quadriceps strength preservation was measured as the difference in maximum voluntary isometric contraction (MVIC) preoperatively and postoperatively.

**Results:**

Seventy-eight patients (G1, *n* = 22; G2, *n* = 26; G3, *n* = 30) met our inclusion/exclusion criteria. Patients with FNB retained significantly lower baseline MVIC at 6 h postoperatively (*p* = 0.001), but there were no differences at 24 and 48 h. There were no differences between the groups in functional outcomes at any time point. Patients in the FNB-G1 presented significant lower pain scores at 6 h (*p* = 0.01), 24 h (*p* = 0.005) and 48 h (*p* = 0.01). The highest cumulative opioid requirement was reported in ACB-G3.

**Conclusion:**

For patients undergoing TKA, FTB and ACB preserve quadriceps strength better than FNB at 6 h postoperatively, but there are no differences at 24 and 48 h. Moreover, this early inferiority does not translate to worse functional outcomes at any time point. FNB is associated with better pain control at 6, 24 and 48 h after surgery, while ACB presents the highest cumulative opioid requirement.

**Clinical trial registration:**

This study was registered in clinicaltrials.gov (NCT03518450; https://clinicaltrials.gov/ct2/show/NCT03518450; submitted March 17, 2018).

## Introduction

Every year, the number of total knee arthroplasties (TKA) is increasing [1]. Acute postoperative pain associated with this procedure has a negative impact for the patient and limits early mobilization and rehabilitation. Therefore, multimodal analgesia plays a central role in patient recovery [2]. Peripheral nerve blocks (PNB) are becoming more frequently used as the application of ultrasound (US) helps anesthesiologists to develop more selective techniques.

The anterior innervation of knee joint seems to be the most relevant for postoperative pain after TKA. The femoral nerve provides the anteromedial sensory innervation of the knee; femoral nerve block (FNB) has been shown to be an effective and well-established analgesic technique for TKA [3]. However, FNB associates quadriceps weakness and potential for an increased risk of falls, which have lead researchers to consider alternative motor-spearing blocks [4]. The femoral triangle block (FTB) and adductor canal block (ACB) have been proposed as an effective alternative for TKA [5]. Their analgesic outcomes have been reported to be similar to FNB but affecting a minimal number of motor fibers, which may allow a faster functional recovery [3, 6]. However, the optimal PNB for TKA is still a matter of debate.

The primary objective of this prospective, double-blinded, randomized controlled trial (RCT) was to compare quadriceps muscle strength preservation between three PNB techniques in TKA: FNB vs FTB vs ACB. The secondary objective was to analyze pain control and functional outcomes.

## Methods

### Study design

This study is a prospective, double-blinded RCT. It was approved by the Ethics Committee (R(AG)299/2017) and it was registered in clinicaltrials.gov (NCT03518450; submitted March 17, 2018). The present trial is reported in compliance with the CONSORT statement.

From April 2018 to April 2019, patients who were listed to undergo an elective, unilateral, primary TKA at the Department of Orthopedic Surgery of our center (a level 1 healthcare institution) were screened for the study. The inclusion criteria were: (a) patients older than 18 years of age, (b) elective, unilateral, primary TKA, (c) operated in our center between April 2018 and April 2019 and (d) programmed to be the first surgery of the day. Exclusion criteria included: (a) revision knee surgery, (b) previous diagnosis of unstable psychiatric pathology, (c) dementia, (d) kidney or hepatic disease that contraindicated the use of NSAIDs and/or paracetamol, (e) allergy to any component of multimodal analgesia, (f) use of opioids greater than 30 mg of oral morphine equivalent daily dose (oMEDD) and g) history of drug abuse.

Eligible patients were interviewed and provided with a printed information sheet. After written informed consent was obtained, the patient was assigned a participant number and the preoperative battery of tests was performed. Then, the participants were randomized into three experimental groups: FNB group (FNB-G1), distal FTB group (FTB-G2) and proximal ACB group (ACB-G3). The randomization and allocation were computer generated by a statistician not otherwise involved in the study. Sequentially numbered opaque envelopes with each participant intervention group were made. Once in the operating theatre, the assigned anesthesiologist opened the corresponding envelope with the designated group. Excluding the anesthesiologist, all other professionals and the patient were blinded to treatment allocation.

### Outcome measures

The primary outcome was quadriceps muscle strength preservation. It was measured as the difference in quadriceps strength preoperatively and 6 h after surgery, using the maximum voluntary isometric contraction (MVIC). The MVIC was assessed by a Physical Medicine and Rehabilitation specialist at the preadmission consult and at 6, 24 and 48 h postoperatively. A handheld dynamometer (HHD) was used (MicroFET2; Hoggan Health Industries Inc., UT, USA) [7]. In a seated position and with the knee flexed at 60º, with the HHD placed perpendicular to the tibial crest 5 cm proximal to the medial malleolus, patients were told to push against the HDD and hold the final position for 3 s. For each assessment, three measurements were taken and the mean value was used for calculations.

The secondary outcomes were pain control and functionality. Pain was assessed preoperatively and at 6, 24 and 48 h after surgery. Pain measurement was obtained at rest and during 45° passive flexion of the knee on a 0 (no pain) to 100 (pain as bad as it can be) visual analogue scale (VAS). Furthermore, cumulative opioid consumption was calculated at these time points using units of morphine milligram equivalents (MME) to analyze supplementary pain relief requirement. Functional tests were also measured preoperatively and at 6, 24 and 48 h postoperatively using the Timed Up and Go test (TUG), 30-secs-Chair-Stand-Test (30′ CST), 10-Point-Mobility-Scale (10-PMS), Daniels test and passive/active Range of Motion (ROM). Length of hospital stay (LOS) was measured from the surgery day until patient discharge.

### Intervention technique

#### Preoperative

All PNB were performed by an experienced anesthesiologist under aseptic conditions, using a high-frequency (10–12 MHz) linear US probe (M-Turbo; SonoSite, Bothell, WA, USA), a 100-mm needle (Echoplex; Vygon, Lansdale, PA, USA) and 30 mL of 0.25% bupivacaine with 4 mg of dexamethasone. Thirty minutes after performing the intervention technique, the efficacy of the nerve block was evaluated by testing for loss to cold sensation in the saphenous nerve innervation area. Then, an IPACK block [8] was administered to all the patients using 30 mL of 0.25% bupivacaine and 4 mg of dexamethasone. Patients underwent spinal anesthesia using a maximum dose of 9 mg of 0.5% bupivacaine and 20 mcg of fentanyl at the L3/L4 intervertebral space. Intravenous midazolam was used to achieve a mild sedation. All patients received dexketoprofen (50 mg, IV) 30 min prior to surgery and paracetamol (1 g, IV) during surgery. Perioperative corticosteroids were not administered.

##### Group one (G1): FNB technique

Placing the US probe on the proximal thigh perpendicular to its long axis, the femoral artery was located and followed towards the inguinal ligament. The femoral nerve was identified external to the artery. The injection point was located 1–2 cm distal to the inguinal ligament before the femoral artery bifurcation. Using an in-plane approach, the needle was advanced in a lateral-to-medial direction until the tip was located next to the femoral nerve.

##### Group two (G2): distal FTB technique

The US probe was placed in a transverse orientation over the mid-thigh. The sartorius muscle was identified and followed to the point where its medial border intersected the adductor longus medial border. Once the proximal end of the AC was identified, the distance from this point to the inguinal ligament was divided in thirds. The femoral artery was followed to the point where the distal and medial third of the FT met. A lateral-to-medial in-plane approach was used to advance the needle and locate its tip in the lateral aspect of the femoral artery.

##### Group three (G3): proximal ACB technique

After the US identification of the proximal end of the AC, the transducer was moved distally to identify the adductor hiatus, where the femoral artery diverges from the sartorius muscle and goes through the adductor magnus tendon. Once the two ends of the AC were located, the distance between them was divided in thirds. The injection point was located where the proximal and medium thirds met. The needle was inserted from the lateral aspect of the thigh until locating the tip laterally to the femoral artery.

#### Intraoperative

During surgery, patients had standard monitors and intravenous access. All surgeries were performed by specialized orthopaedic surgeons from the knee unit. Patients were placed in the supine position. A medial parapatellar approach, under tourniquet, was used to implant a tricompartmental prosthesis (TKA). Local infiltration of analgesia (LIA) was not used.

#### Postoperative

After surgery, all patients were transferred to the post-anesthesia care unit (PACU), where they remained until meeting the discharge criteria. Postoperative pain was assessed every 15 min. Patients with VAS scores of 40/100 or higher were managed with 2–4 mg of IV morphine every 10 min as needed. If the patient continued experiencing severe pain at the anterior aspect of the knee after the administration of 0.2 mg/kg of morphine within the first 6 h post-surgery, the PNB would be considered to have failed and a rescue FNB would be performed.

Once out of the PACU, for the first 48 postoperative hours, the analgesic regimen consisted of 50 mg of IV dexketoprofen every 8 h and 1 g of IV paracetamol every 8 h. If the patient requested more analgesics and VAS score was 40/100 or higher, 1 mg/kg of IV tramadol was administered and repeated every 8 h if needed. IV morphine (2 mg/h) was used as second line rescue agent.

### Statistical analysis

The primary endpoint of this study was quadriceps muscle strength preservation (MVIC). In order to have an 80% chance of detecting 1.5 units of differences, with α = 0.05, a sample size of 38 participants per treatment group was needed for this study. Assuming a 10% dropout rate, a total sample size of 126 subjects (42 per group) was planned.

The Kolmogorov–Smirnov test was used to assess normality. Normally distributed data were reported as mean (standard deviation [SD]). Non-normally distributed and ordinal data were reported as median (inter-quartile range [IR]), and categorical data were presented as number (percentage [%]). To study the possible relationship between categorical variables, Pearson *X*^2^ test or Fisher’s exact test were used as appropriate. To study the relationship between continuous data, Student’s *T* test or Mann–Whitney *U* test were used. The Kruskal–Wallis test was used to compare outcomes between the three groups. When significant differences were found, the test was followed by pairwise comparison using the Dunn’s multiple comparisons post hoc test with Bonferroni correction for multiple tests (three comparisons).

Analysis of data was performed by intention to treat analysis. All *p*-values were two-tailed and a *p*-value < 0.05 was considered statistically significant. Statistical analysis was performed using SPSS 14.0 (IBM Corp., Armonk, New York, USA).

## Results

**Fig. 1:**
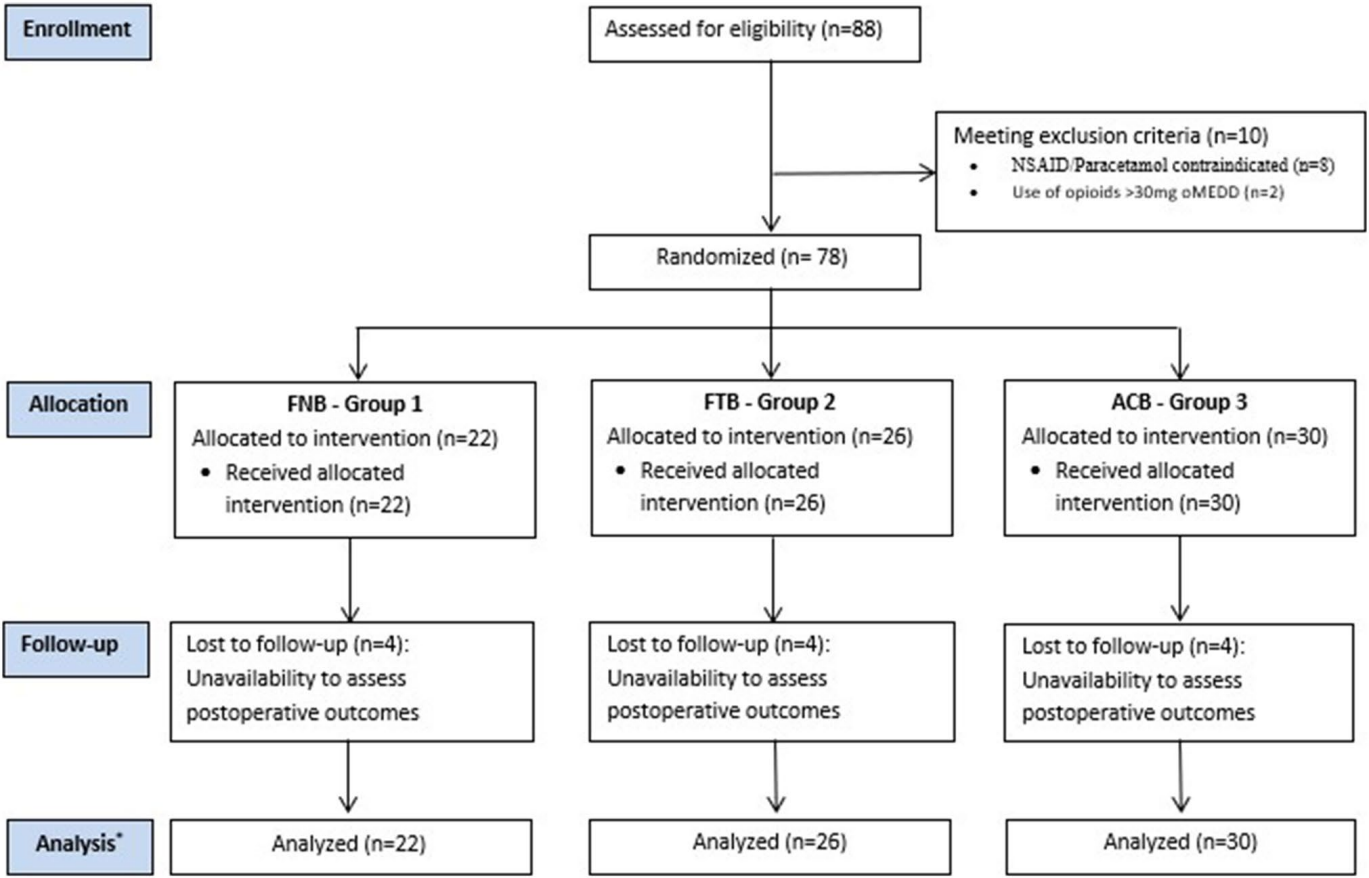
CONSORT diagram. Boxes in the left represent the committed step of the clinical trial. * Analysis of data was performed by intention to treat analysis. *FNB* femoral nerve block; *FTB* femoral triangle block; *ACB* Adductor canal block

Seventy-eight patients met our inclusion/exclusion criteria and were recruited for the study. An interim analysis was performed and significant differences in analgesic outcomes were noticed; recruitment was stopped as it was unethical to continue. The CONSORT diagram is presented in Fig. 1. Twelve participants (G1, *n* = 4; G2, *n* = 4; G3, *n* = 4) were considered lost to follow-up due to unavailability to assess their postoperative outcomes. The three groups were comparable in terms of baseline demographic data (Table 1), except for a higher median age in G1 (G1 = 78.5 vs G2 = 70 vs G3 = 73.5 years; *p* = 0.02).

**Table 1:** Demographics baseline characteristics

Variable	Group 1 (*n* = 22)	Group 2 (*n* = 26)	Group 3 (*n* = 30)	*p*-Value
Age (years), median (IR^a^)	78.5 (9)	70 (14)	73.5 (8)	0.02*
Sex (female), *n* (%)	12 (66.7)	13 (59)	22 (84.6)	0.1
Body mass index (kg/m^2^), median (IR^a^)	30.5 (9)	30.5 (7)	32 (8)	0.06
Tourniquet time (min), median (IR^a^)	82 (44)	67.5 (26)	63 (20)	0.08

^*^Statistically significant differences

^a^*IR* interquartile range

The quadriceps strength preservation assessment (Table 2) showed that subjects in G1 retained a significantly lower percentage of baseline MVIC at 6 h postoperatively in comparison with the other two groups (*p* = 0.001). However, there were no significant differences at 24 and 48 h.

**Table 2:** Main outcomes measures at each time point and comparison of the three groups (FNB-G1, FTB-G2 and ACB-G3)

Variable	Time point (h)	G1 (*n* = 22)	G2 (*n* = 26)	G3 (*n* = 30)	*p*-Value
Preserved MVIC (%)	6	15.4 (40)	41.4 (36.7)	40 (42.8)	0.001*
	24	30 (43.3)	43.1(36.3)	43.6 (30.2)	0.5
	48	40 (38)	39.4 (36.9)	43.6 (40)	0.7
Pain at rest (VAS, 0–100)	6	10 (20)	15 (50)	55 (60)	0.01*
	24	5 (40)	45 (60)	55 (20)	0.005*
	48	25 (50)	50 (40)	55 (30)	0.01*
Pain at 45º passive flexion (VAS, 0–100)	6	30 (50)	55 (60)	70 (60)	0.006*
	24	50 (70)	65 (40)	80 (30)	0.09
	48	50 (30)	80 (40)	80 (30)	0.11
Morphine consumption (mg·kg^−1^)	6	0 (0)	0 (0.06)	0.09 (0.15)	0.001*
	24	0 (0)	0.05 (0.10)	0.09 (0.14)	0.01*
	48	0.02 (0.10)	0.02 (0.12)	0.1 (0.30)	0.1

Results are reported as median and interquartile range: median (IR). Kruskal–Wallis test was performed to compare the groups

^*^Statistically significant differences

**Fig. 2:**
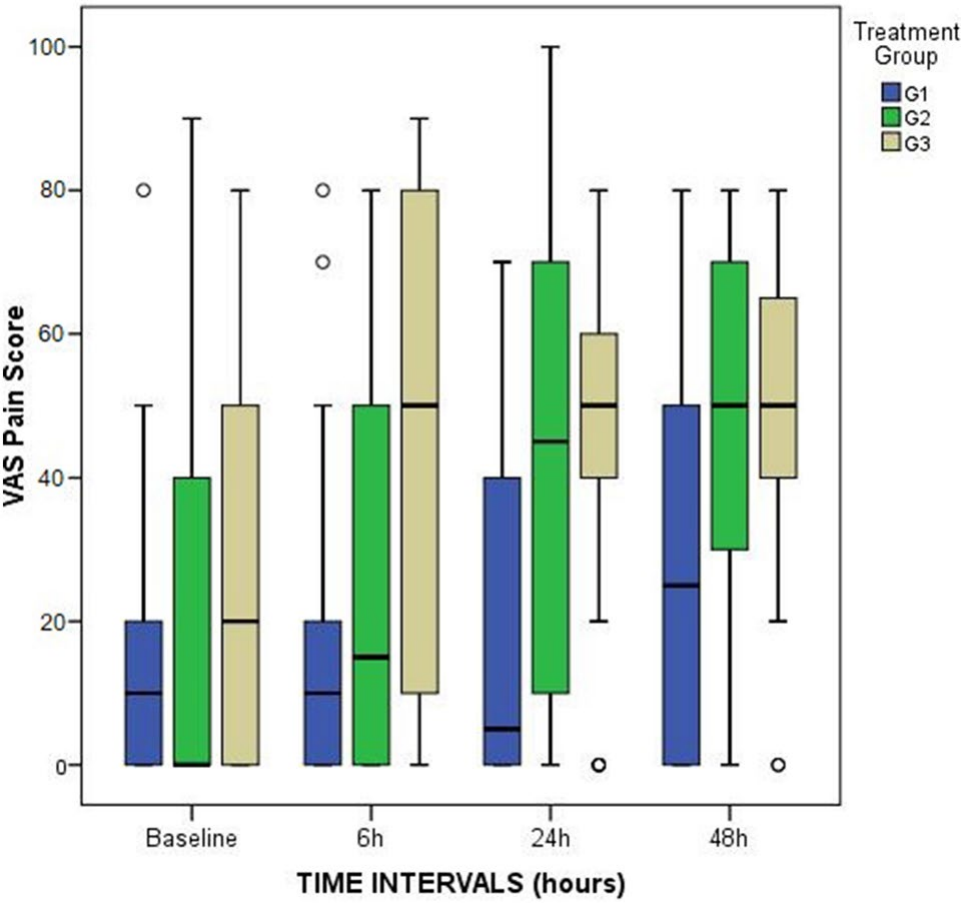
Box and whiskers plot showing pain at rest scores (VAS: Visual Analog Scale) at each time point and comparison of the three groups (FNB-G1, FTB-G2 and ACB-G3)

**Fig. 3:**
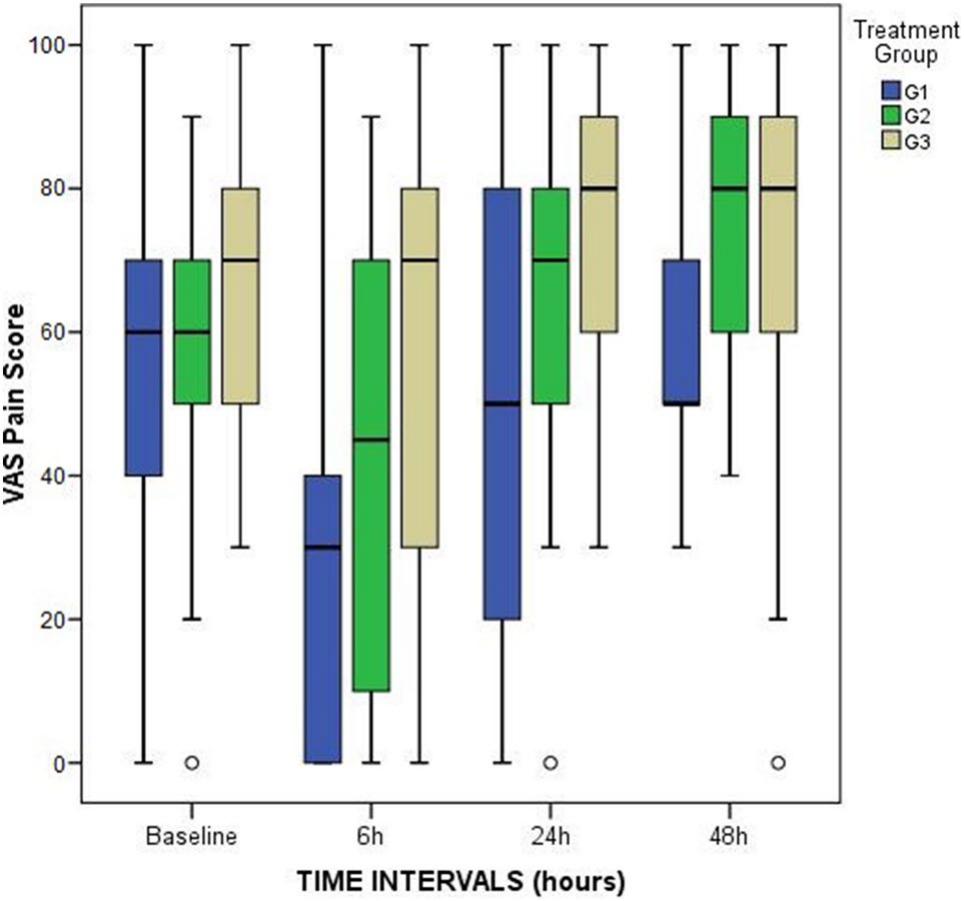
Box and whiskers plot showing pain at 45° passive knee flexion scores (VAS: Visual Analog Scale) at each time point and comparison of the three groups (FNB-G1, FTB-G2 and ACB-G3)

There were no observed group differences in VAS pain scores before surgery. However, patients in the FNB-G1 presented statistically significant lower median pain scores during rest at 6 h (*p* = 0.01), 24 h (*p* = 0.005) and 48 h (*p* = 0.01) (Fig. 2). The biggest difference between the three groups occurred at 24 h postoperatively: FNB-G1, 5 (IR: 40); FTB-G2, 45 (IR: 60); ACB-G3, 55 (IR, 20). Patients in G1 also referred lower pain with passive knee flexion at 6 h (*p* = 0.006), (Fig. 3). No pairwise comparisons were found to be significant between G1 and G2. On the other hand, patients from G1 when compared to G3, presented significant lower pain at rest at 6 (*p* = 0,02), 24 (*p* = 0.005) and 48 h (*p* = 0.02), and at 6 h for passive flexion (*p* = 0.005).

**Fig. 4:**
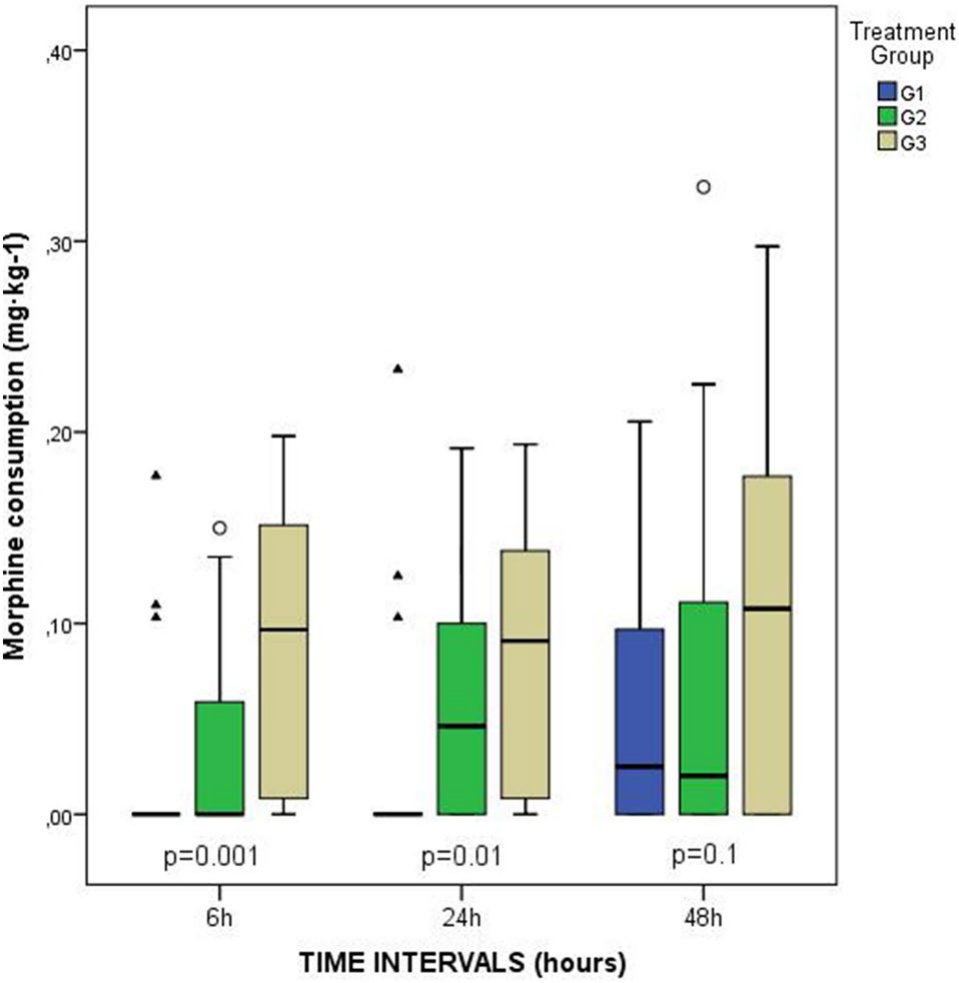
Box and whiskers plot showing the cumulative opioid consumption (morphine milligram equivalents) at each time point and comparison of the three groups (FNB-G1, FTB-G2 and ACB-G3)

Pain medication analysis showed that opioid requirement in G3 was significantly higher at 6 h (*p* = 0.001) and 24 h (*p* = 0.01), (Fig. 4). Moreover, the overall cumulative dose of MME was also higher in G3: FNB-G1, 0.02 (IR: 0.23); FTB-G2, 0.07 (IR: 0.17); ACB-G3, 0.28 (IR, 0.27) mg·kg^−1^; *p* = 0.004.

There were no significant differences between the groups in functional test outcomes (time needed to perform the TUG test, 30′ CST, 10-PMS and Daniels test compared to the baseline value at each time point), (Table 3). There were no differences neither in active/passive knee ROM, except for active knee flexion at 6 h (G3 presented the worst outcomes). Lengths of hospital stay between the three groups were comparable: FNB-G1, 5.5 (IR: 3); FTB-G2, 5.5 (IR: 3); ACB-G3, 68 (IR, 4) days; *p* = 0.9.

**Table 3:** Functional measures at each time point and comparison of the three groups (FNB-G1, FTB-G2 and ACB-G3)

Variable median (IR^a^)	Time point (h)	G1 (*n* = 22)	G2 (*n* = 26)	G3 (*n* = 30)	*p*-Value
Active ROM, flexion (degrees)	6	− 30 (25)	− 22.5 (62.5)	− 53.7 (50)	0.01*
	24	− 15 (44)	− 22.5(48.7)	− 27.5 (45)	0.09
	48	− 20 (20)	− 10 (32.5)	− 22.5 (31.2)	0.7
Active ROM, extension (degrees)	6	0 (15)	0 (13.7)	0 (13.7)	0.9
	24	0 (0)	5 (13.7)	5 (15)	0.8
	48	0 (10)	5 (17.5)	3.5 (21.2)	0.7
Passive ROM, flexion (degrees)	6	− 20 (55)	− 35 (61.2)	− 42.5 (50)	0.2
	24	− 30 (50)	− 27.5(35)	− 37.5 (40)	0.3
	48	− 35 (65)	− 17.5 (18.7)	− 22.5 (40)	0.4
Passive ROM, extension (degrees)	6	5 (20)	5 (10)	0 (13.7)	0.4
	24	0 (15)	0 (8.7)	5 (17.5)	0.5
	48	5 (15)	0 (15)	0 (17.5)	0.8
Daniels test (muscle contraction level)	6	− 2 (8)	0 (1.7)	− 1 (2.7)	0.05
	24	− 2 (2)	− 1(3)	− 1.5 (2)	0.06
	48	− 2 (2)	− 1.5 (4)	− 1.5 (2)	0.6
TUG test (s)	6	11 (9)	12 (17.5)	12.5 (18.2)	0.7
	24	11 (9)	9 (18.5)	14 (7.5)	0.3
	48	9 (23)	2.5 (34)	10.5 (32)	0.7
30’ CST test (number of stands)	6	− 7 (9)	− 6 (9.5)	− 4 (7.7)	0.09
	24	− 5 (11)	− 6 (9.2)	− 4 (3.7)	0.4
	48	− 5 (11)	− 2.5 (8.2)	− 3.5 (3.5)	0.2
10 PMS test (points)	6	− 6 (6)	− 6 (7.7)	− 7.5 (6.5)	0.3
	24	− 5 (4)	− 6 (6.5)	− 5.5 (3)	0.9
	48	− 3 (3)	− 3.5 (5)	− 3.5 (3.5)	0.8
Hospital stay (days)	–	5.5(3)	5.5 (3)	6 (4)	0.9

^*^ Statistically significant differences

^a^ *IR* Interquartile Range

Outcomes have been presented as the difference to the baseline value. Results are reported as median and interquartile range: median (IR). Kruskal–Wallis test was performed to compare the groups

## Discussion

The most important finding of this study was that distal FTB and proximal ACB preserve quadriceps muscle strength better than FNB at 6 h postoperatively. However, there were no significant differences between the three blocks in functional outcomes and patients who received a FNB showed better pain control.

The quadriceps strength loss of the FNB-G1 was significantly higher at 6 h postoperatively, but there were no differences between the three groups at 24 or 48 h. Moreover, this initial difference in strength preservation did not translate to worse clinical outcomes. No significant differences were found in functional test outcomes at any time point nor LOS. Most of the authors have also reported worse quadriceps strength preservation with the use of FNB [6, 9–11]. However, different to us, some articles defended that FNB was associated with delayed functional recovery [6, 11, 12]. Based on our experience, despite the FNB was actually associated with an initial higher quadriceps strength loss, it seems not to be of enough magnitude to limit the early functionality.

The ACB has been proposed as an emerging effective analgesic technique in TKA [5]. However, we found that this PNB was associated with the highest cumulative opioid requirement during the postoperative period. Furthermore, patients who received a FNB presented the lowest median pain scores at 6, 24 and 48 h. In the literature, there are mixed results regarding the analgesic efficacy of the ACB. Some authors defend that this technique provides similar outcomes in pain control and opioid requirements than the standard FNB [6, 9, 10, 12]. However, a recent RCT also reported that ACB does not provide equivalent analgesic efficacy to FNB [13]; they highlighted that studies comparing various PNB in TKA are warranted.

Our inferior analgesic outcomes with the use of the ACB may be explained by the absence of a consistent technique among the literature to perform this block. Initially, surface anatomical landmarks were used to locate the ACB injection point, but that was shown to be inaccurate as it usually leaded to the femoral triangle [14]. Later publications were based on US guided landmarks, but confusing the contents and limits of the proximal adductor canal with the distal femoral triangle and, therefore, some proximal ACB were likely to be distal FTB in fact [15]. In the authors’ opinion, clearly defined anatomic and US landmarks are fundamental for anesthetic regional techniques. Both ends of the AC can be precisely identified, and they have been used in recent cadaveric studies [14, 16, 17]. The proximal end of this musculoaponeurotic tunnel is located at the point where the medial border of the sartorius intersects the medial border of the adductor longus. The distal end is found at the adductor hiatus where the femoral artery diverges from the sartorius and goes through the adductor magnus tendons. Houssain et al. [18] noted that varying the locations of the ACB injection point in each study made difficult to draw strong conclusions.

Clinical differences due to multiple ACB locations could be partially explained by the nerve of the vastus medialis (NVM). This branch of the femoral nerve importantly contributes to the innervation of the anteromedial knee joint [17]. Burckett-St Laurant et al. [17], in an anatomic study, detected only the 35% of the extramuscular NVM branches within the AC. Tran et al. [19] found that anterior branches of NVM did not enter the AC and that the posteromedial branch of NVM was separated from the contents of the AC by a thin fascia. Furthermore, some authors have reported the lack of proximal spread of local anesthetic in ACB, with the consequent sparing of the NVM. A cadaveric study by Johnston et al. [16] showed that a 20 mL injection in the distal AC did not spread proximally to the FT and spared the NVM. However, the same volume of dye injected in the distal FT stained the NVM in the 100% of cases. In our study, we used a 30 mL volume for all the groups to ensure that the maximum numbers of nerve branches were blocked [20], to homogenize the three groups and to compare our outcomes with similar trials. In addition, the iPACK block was administered to all participants to provide posterior knee capsule analgesia to the three study groups [8]. We aimed to avoid a possible confounding variable when comparing pain scores and opioid consumption.

In our study, we did not use perioperative corticosteroids nor LIA. Both of them could be useful tools to enhance pain control in a multimodal analgesia setting. It has been reported that intravenous dexamethasone reduces postoperative pain, opioid consumption and nausea [21]. However, it is controversial which number of doses, dosage, and frequency should be recommended. Corticosteroids may increase blood glucose levels and should be used with caution in patients with diabetes. Furthermore, there is insufficient evidence on whether intravenous corticosteroids increase the risk of periprosthetic joint infection or wound-healing complications [21]. On the other hand, LIA has demonstrated to reduce postoperative pain without affecting motor function. Therefore, it can be useful to facilitate early ambulation and rehabilitation. However, it is not clear if LIA, PNB or its combination present superior analgesic properties [22].

Our study is subjected to several limitations. First, an interim analysis detected significant differences in analgesic outcomes between the three groups and recruitment was stopped. Therefore, the minimum sample size was not reached. The interim analysis was not planned in advance; however, it was carried out due to an apparent highly heterogeneity in pain control among patients. Second, there was an uneven distribution of participants between the three groups. It could have been better controlled by using a block randomization design. However, the study groups were comparable in terms of baseline demographic data (we consider that the higher median age in G1 was statistically significant but clinically not relevant). Third, the study did not contain a control group without PNB (the local analgesic effect could be provided by LIA). On the other hand, it is a double-blinded RCT, which may allow to provide the highest level of evidence. Another strength of our work is that all the PNB were performed by an experienced anesthesiologist, using strict injection point location through anatomic and US landmarks. Furthermore, to homogenize the three groups, we used a 30 mL volume for all the PNB and an iPACK block was added to all participants.

## Conclusion

For patients undergoing TKA, distal FTB and proximal ACB preserve quadriceps strength better than FNB at 6 h postoperatively, but there are no differences at 24 and 48 h. Moreover, this early inferiority in strength preservation do not translate to worse functional outcomes at any time point nor longer LOS. FNB is associated with better pain control at 6, 24 and 48 h after TKA surgery, while ACB presents the highest cumulative opioid requirement. More RCTs comparing multiple PNB are necessary to clearly elucidate the role of each block in the TKA surgery.

## Data Availability

The manuscript has no associated data or the data will not be deposited.
